# Tart Cherry Extracts Reduce Inflammatory and Oxidative Stress Signaling in Microglial Cells

**DOI:** 10.3390/antiox5040033

**Published:** 2016-09-22

**Authors:** Barbara Shukitt-Hale, Megan E. Kelly, Donna F. Bielinski, Derek R. Fisher

**Affiliations:** USDA-ARS, Human Nutrition Research Center on Aging, Tufts University, 711 Washington Street, Boston, MA 02111, USA; megan.kelly@ars.usda.gov (M.E.K.); donna.bielinski@tufts.edu (D.F.B.); derek.fisher@ars.usda.gov (D.R.F.)

**Keywords:** antioxidant, anti-inflammatory, polyphenols, anthocyanins, cytokines

## Abstract

Tart cherries contain an array of polyphenols that can decrease inflammation and oxidative stress (OS), which contribute to cognitive declines seen in aging populations. Previous studies have shown that polyphenols from dark-colored fruits can reduce stress-mediated signaling in BV-2 mouse microglial cells, leading to decreases in nitric oxide (NO) production and inducible nitric oxide synthase (iNOS) expression. Thus, the present study sought to determine if tart cherries—which improved cognitive behavior in aged rats—would be efficacious in reducing inflammatory and OS signaling in HAPI rat microglial cells. Cells were pretreated with different concentrations (0–1.0 mg/mL) of Montmorency tart cherry powder for 1–4 h, then treated with 0 or 100 ng/mL lipopolysaccharide (LPS) overnight. LPS application increased extracellular levels of NO and tumor necrosis factor-alpha (TNF-α), and intracellular levels of iNOS and cyclooxygenase-2 (COX-2). Pretreatment with tart cherry decreased levels of NO, TNF-α, and COX-2 in a dose- and time-dependent manner versus those without pretreatment; the optimal combination was between 0.125 and 0.25 mg/mL tart cherry for 2 h. Higher concentrations of tart cherry powder and longer exposure times negatively affected cell viability. Therefore, tart cherries (like other dark-colored fruits), may be effective in reducing inflammatory and OS-mediated signals.

## 1. Introduction

Oxidative stress (OS) and inflammation in the brain contribute to the decline of motor abilities and cognitive performance with age [1,2]. While OS and inflammation both increase with age, the body’s ability to defend and repair itself decreases through the lifespan [3,4]. This makes the brain more susceptible to the deleterious effects of OS and inflammation. Increased intake of antioxidants and anti-inflammatory compounds are believed to protect against this decline [5]. In this regard, certain foods with antioxidant and anti-inflammatory effects have been shown to help protect against the negative effects of aging.

Polyphenols are compounds from plants involved in antioxidant and anti-inflammatory cell activities that may be responsible (via an array of health-related bioactivities) for the multitude of beneficial effects that have been reported due to fruit and vegetable consumption [6]. There are thousands of different polyphenols found in plants, which are categorized into several groups based on their unique molecular structure. The presence of a number of bioactive compounds, including polyphenols, suggest tart cherries as a potential nutritional therapeutic to curtail the negative effects of aging. Tart cherries are rich in anthocyanins (one class of polyphenols), with cyanidin being the most abundant [7,8], as well as flavan-3-ols and flavonols [9]. The components of cherries may act directly to improve brain cell function and signaling, and/or may be more generally affecting extra-neuronal parameters of survival—such as inflammation—within the aging brain to improve behavior. In addition, cherries have been shown to reduce inflammation [10,11,12,13] and decrease oxidative stress [14,15,16].

Research with tart cherries and other dark-colored fruit has shown that their polyphenols become available to humans and rats in the bloodstream after consumption [17]. Consumption increases the levels of antioxidants and anti-inflammatory compounds due to the polyphenols from the tart cherries. Furthermore, it has been shown that cherry anthocyanins accumulate in the brain of young rats after 3 weeks of feeding with either 1% or 10% tart cherry-supplemented diets in a dose-dependent manner [18], and that dietary supplementation of tart cherries to rats aged 19–21 months improves age-related deficits in behavioral and neuronal functioning [19]. Similar age-related improvements were found when diets were supplemented with other dark-colored fruits, such as blueberry, strawberry, spinach, blackberries, cranberries, black currants, and Concord grape juice [20,21,22,23,24].

When lipopolysaccharide (LPS) is applied to microglial cells, it activates the cells in the same way as what occurs during a bacterial infection, which increases OS and inflammation. Increased inflammation leads to increased levels of inflammatory stress signals, such as nitric oxide (NO), tumor necrosis factor-alpha (TNF-α), and cyclooxygenase-2 (COX-2), as well as increased expression of inducible nitric oxide synthase (iNOS). A previous study using these markers showed the anti-inflammatory and antioxidant effects of pretreatment of BV-2 mouse microglial cells with açai pulp fractions, which are also rich in anthocyanins [25]. After açai pretreatment, the cells were treated with LPS. These cells showed reduced production of NO, TNF-α, and COX-2, and reduced iNOS expression relative to cells not pretreated with açai extracts, showing that dark-colored fruits rich in polyphenols can protect against OS and inflammation in microglial cells.

The present study sought to determine whether the application of tart cherry to HAPI rat microglial cells would protect against LPS-induced OS and inflammation in a similar fashion. To more closely relate to our behavioral study with tart cherry [19], we used HAPI cells in these experiments because they are derived from rats, as opposed to the BV-2 cells, which are derived from mice. HAPI rat microglial cells were pretreated for various durations and concentrations with tart cherry powder. After subsequent treatment with LPS, OS and inflammation were determined by measuring levels of NO, TNF-α, COX-2, and iNOS. Furthermore, this study sought to determine the ideal tart cherry concentration and pretreatment time to minimize OS and inflammation.

## 2. Materials and Methods

### 2.1. Cell Culture

HAPI rat microglial cells (generously provided by Dr. Grace Sun, University of Missouri, Columbia, MO, USA) were maintained in Dulbecco’s modified Eagle’s medium (DMEM, Invitrogen, Grand Island, NY, USA) supplemented with 10% fetal bovine serum (FBS), 100 U/mL penicillin, and 100 ug/mL streptomycin at 37 °C in a humidified incubator under 5% CO_2_. Cells were grown in 100 mm plates and then split into 12-well plates prior to treatment. All treatment groups were assayed in duplicate. For experiments, cells were incubated in DMEM without phenol red. Cells were then pretreated with Montmorency tart cherry powder (0.062, 0.125, 0.250, 0.500, 1.000 mg/mL) diluted in media, or with control media for 1, 2, or 4 h. After the pretreatment, the media was removed and cells were stimulated overnight with lipopolysaccharide (LPS, Sigma-Aldrich, St. Louis, MO, USA) at 100 ng/mL or 0 ng/mL in DMEM without phenol red. The freeze-dried Montmorency tart cherry powder (*Prunus cerasus* L.*)* was provided by the Cherry Marketing Institute (Dewitt, MI, USA), and its polyphenolic composition has been previously published [9,26]. Additionally, HAPI cells have been used in previous studies to study the effects of LPS-induced inflammation and possible neuroprotection [27,28,29].

### 2.2. Viability

Cell viability was measured using Live/Dead Cellular Viability/Cytotoxicity Kit (Molecular Probes, Eugene, OR, USA). Calcein AM labels live cells with intact membranes with a green fluorescent compound. Ethidium homodimer-1 labels dead cells with damaged membranes with red fluorescence. Fluorescent images of the cell were captured with a Nikon TE2000U inverted fluorescent microscope.

### 2.3. Nitrite Quantification

Under physiological conditions, NO is oxidized into nitrite (NO_2_^-^). Therefore, to assess the production of NO from HAPI cells after the pretreatments and treatments described above, the extracellular release of nitrite was measured by Greiss reagent (Invitrogen) according to manufacturer’s instructions. Absorbance was read at 548 nm, and the concentration of nitrite was calculated with the linear equation derived from the standard curve generated by known concentrations of nitrite.

### 2.4. Western Blots

Cells were washed in ice-cold PBS, re-suspended, and lysed by agitation in CelLytic-M Cell Lysis Reagent (Sigma). Cells were then centrifuged at 18,000× *g* for 10 min at 4 °C. The resultant supernatant lysate was used for blotting, and the pellet was discarded. Western blots were performed as described by Poulose and colleagues [25], except that 10% polyacrylamide gels were used. Primary antibodies for iNOS (Millipore, Billerica, MA, USA) and COX-2 (Santa Cruz, Dallas, TX, USA) were used at 1:1000 dilutions for incubation overnight at 4 °C. Following enhanced chemiluminescence (ECL) development, the optical density of antibody-specific bands was analyzed by the LabWorks image acquisition and analysis software (UVP, Upland, CA, USA).

### 2.5. TNF-α ELISA

Quantification of tumor necrosis factor-alpha (TNF-α) in cell-conditioned media was performed with an enzyme-linked immunosorbent assay (ELISA, eBioscience, San Diego, CA, USA) according to the manufacturer’s instructions. TNF-α concentration for each sample was calculated from the linear equation derived from the standard curve of known concentrations of the cytokine.

### 2.6. Data Analysis

All statistical analyses were performed using SYSTAT software (SPSS, Inc., Chicago, IL, USA). Data are expressed as mean ± SEM. The data were analyzed by three way analyses of variance (ANOVA) with tart cherry dose, duration, and LPS exposure as experimental factors, followed by post hoc testing with Tukey’s HSD test to determine differences among groups. Results were considered statistically significant if the observed significance level *p* was <0.05. Note that for each dependent measure, those cells treated with LPS alone were statistically higher than the control conditions without LPS, which were not different (data not shown for the no LPS condition). Additionally, pretreatment with tart cherry did not significantly affect cells in the absence of LPS in any of the endpoints assayed (data not shown).

## 3. Results

### 3.1. Viability

**Figure A1:**
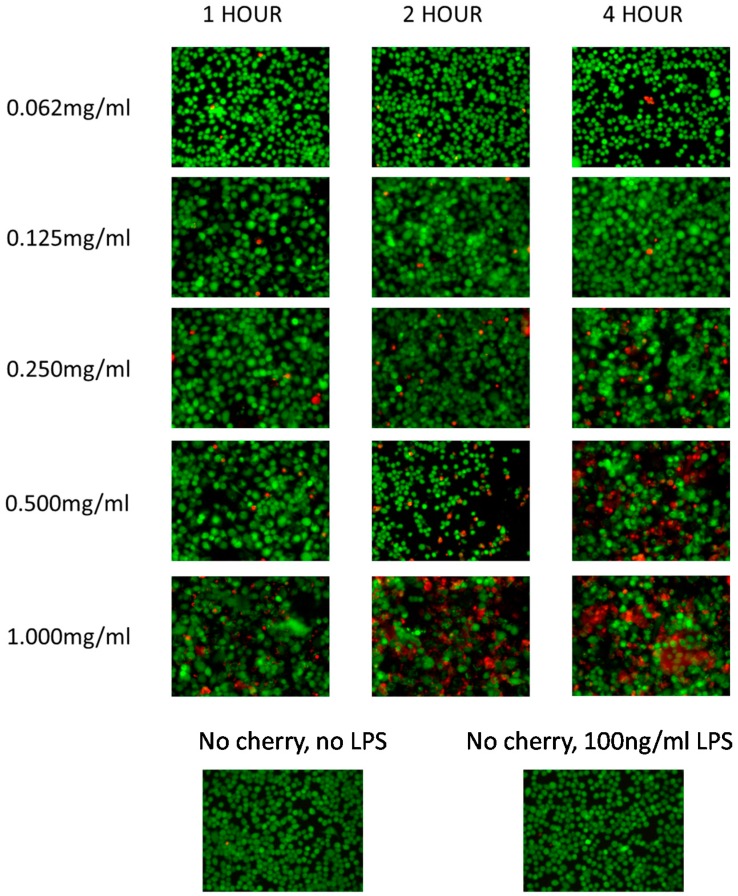
Effect of tart cherry powder on cellular viability. Representative images of cells treated with tart cherry powder (0.062, 0.125, 0.250, 0.500, or 1.000 mg/mL) for 1, 2, or 4 h, as well as cells treated with 0 cherry and 0 or 100 ng/mL LPS. Live cells with intact membranes are labeled green with Calcein AM, while dead cells with damaged membranes are labeled red with ethidium homodimer-1. Images were taken under a fluorescent microscope at 80× magnification.

Higher concentrations of tart cherry powder and longer exposure times led to increased cell death. The images (see Appendix, Figure A1) showed that 1.000 mg/mL of tart cherry powder was too high of a concentration for treatment, and therefore only the lower concentrations of 0.062, 0.125, 0.250, and 0.500 mg/mL were used for testing protection against LPS-induced inflammation and OS. The 0.500 mg/mL dose at 2 and 4 h also caused minor decreased viability. There was no change in viability in control cells (a dose of 0 cherry and 0 LPS) or cells treated with 100 ng/mL LPS, showing that this dose of LPS did not result in cell death, as has been shown previously [29].

### 3.2. Nitric Oxide

**Figure 1:**
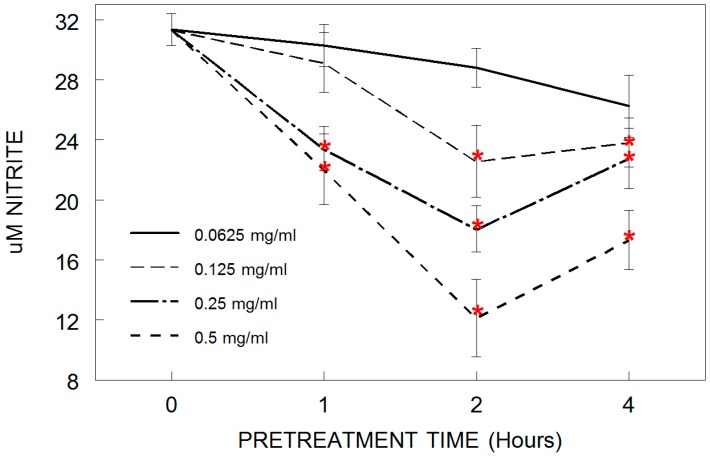
Production of extracellular nitric oxide (NO) in HAPI microglial cells pretreated with tart cherry powder (0.062, 0.125, 0.250, or 0.500 mg/mL) for 0 (LPS only/alone control), 1, 2, or 4 h, and stimulated overnight with lipopolysaccharide (LPS, 100 ng/mL). Data are expressed as mean ± SEM and quantified using the Greiss reagent. Each tart cherry pretreatment was compared against LPS treatment alone. Comparative post hoc analyses were made by Tukey’s HSD with significance at (*) *p* < 0.05 versus LPS.

Results showed that tart cherry pretreatment attenuated LPS-induced nitric oxide (NO) production in HAPI microglia in a dose- and time-dependent manner (Figure 1). NO is a free radical that can also act as a second messenger involved in a number of functions, including cellular immune response and the activation of apoptosis. LPS application significantly increased nitrite release in all groups (*p* < 0.05 compared to control conditions without LPS). However, LPS-induced nitrite release was significantly (*p* < 0.05) reduced by pretreatment with doses of 0.250 and 0.500 mg/mL cherry at 1 h, and doses of 0.125, 0.250, and 0.500 mg/mL cherry at 2 and 4 h, compared to LPS alone. This decrease in NO release at doses of 0.500 mg/mL at 2 and 4 h, however, is most likely due to the decreased viability at this concentration and time, as shown previously.

### 3.3. iNOS

**Figure 2:**
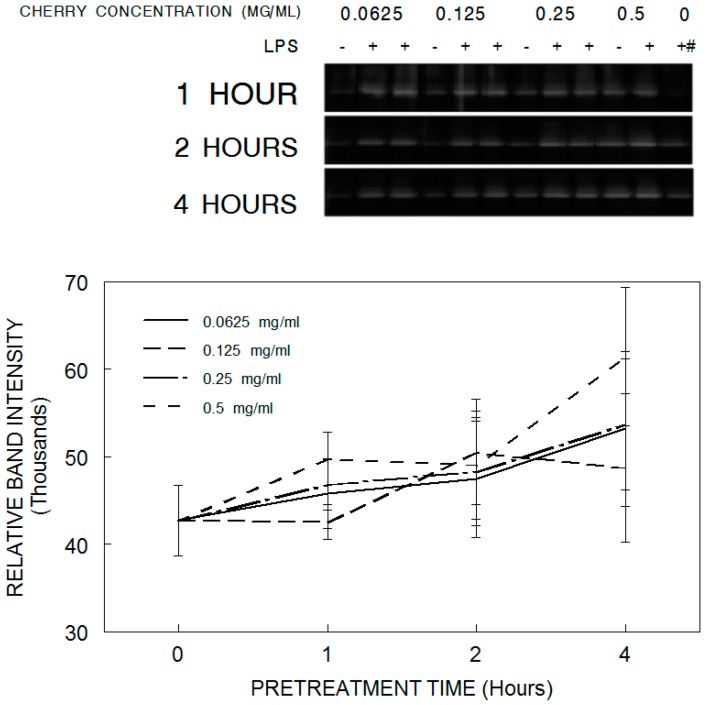
Expression of inducible nitric oxide synthase (iNOS) in HAPI microglial cells pretreated with tart cherry powder (0.062, 0.125, 0.250, or 0.500 mg/mL) for 0 (LPS only/alone control), 1, 2, or 4 h and stimulated overnight with lipopolysaccharide (LPS, 100 ng/mL). Quantitative measurements were made based on the Western blots, and are expressed as mean ± SEM for the immunoreactive band density. Each tart cherry pretreatment was compared against LPS treatment alone, and comparative post hoc analyses were made by Tukey’s HSD. ^#^ column represents blots for 0 cherry with either no LPS (in the 1 h row) or LPS (in the 2 and 4 h row).

Inducible nitric oxide synthase (iNOS) produces the inflammatory mediator nitric oxide (NO). LPS application increased iNOS expression in all groups compared to control conditions not exposed to LPS (*p* < 0.05). However, tart cherry pretreatment did not protect against these LPS-induced increases at any concentration or exposure time (Figure 2).

### 3.4. TNF-α

**Figure 3:**
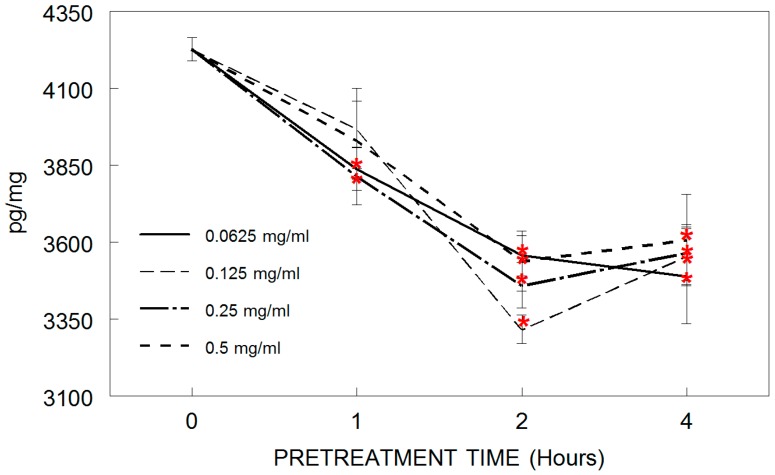
Suppression of tumor necrosis factor-alpha (TNF-α) release in HAPI microglial cells pretreated with tart cherry powder (0.062, 0.125, 0.250, or 0.500 mg/mL) for 0 (LPS only/alone control), 1, 2, or 4 h and stimulated overnight with lipopolysaccharide (LPS, 100 ng/mL). Data are expressed as mean ± SEM (pg/mg of media) as assayed by enzyme-linked immunosorbent assay (ELISA). Each tart cherry pretreatment was compared against LPS treatment alone. Comparative post hoc analyses were made by Tukey’s HSD with significance at (*) *p* < 0.05 versus LPS.

Tart cherry pretreatment reduced the LPS-induced release of the inflammatory cytokine tumor necrosis factor-alpha (TNF-α) in HAPI microglia in a dose- and time-dependent manner (Figure 3). LPS application increased TNF-α release in all groups compared to control groups with no LPS (*p* < 0.05). This increase was significantly reduced by pretreatment with concentrations of 0.062 and 0.250 mg/mL of tart cherry for 1 h, and all doses of cherry at 2 and 4 h, compared to LPS alone. At 4 h, however, TNF-α levels began to rise relative to the 2 h pretreatment, suggesting that 4 h was too long a duration for pretreatment.

### 3.5. COX-2

**Figure 4:**
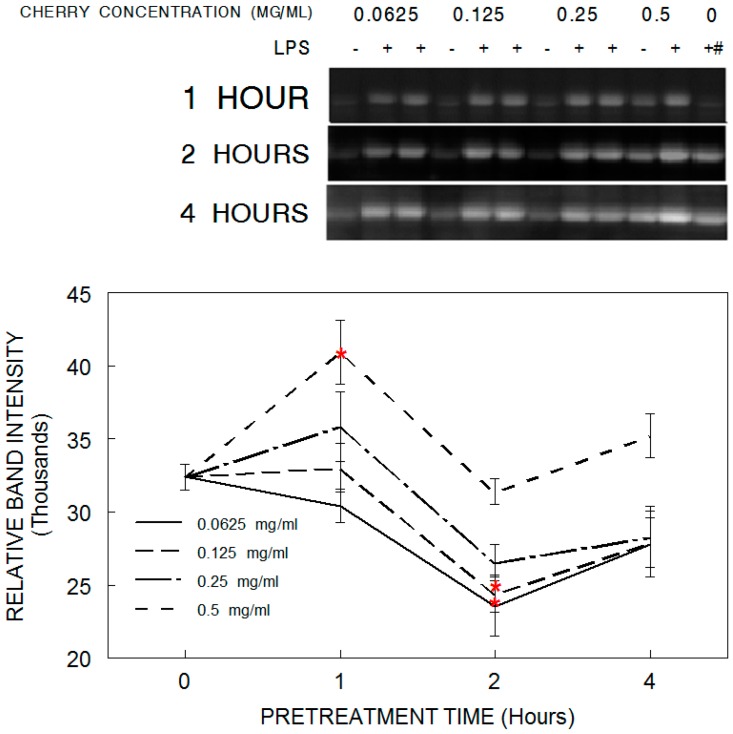
Reduction in cyclooxygenase-2 (COX-2) expression in HAPI microglial cells pretreated with tart cherry powder (0.062, 0.125, 0.250, or 0.500 mg/mL) for 0 (LPS only/alone control), 1, 2, or 4 h and stimulated overnight with lipopolysaccharide (LPS, 100 ng/mL). Quantitative measurements were made based on the Western blots, and are expressed as mean ± SEM for the immunoreactive band density. Each tart cherry pretreatment was compared against LPS treatment alone. Comparative post hoc analyses were made by Tukey’s HSD with significance at (*) *p* < 0.05 versus LPS. ^#^ column represents blots for 0 cherry with either no LPS (in the 1 h row) or LPS (in the 2 and 4 h row).

Results showed that lower doses of tart cherry pretreatment reduced LPS-induced cyclooxygenase-2 (COX-2) expression in HAPI microglia at shorter durations (Figure 4). COX-2 is responsible for the formation of prostanoids, which are inflammatory mediators. LPS application increased COX-2 expression in all groups compared to control conditions not exposed to LPS (*p* < 0.05). This increase was significantly reduced with doses of 0.062 and 0.125 mg/mL cherry at 2 h, compared to LPS alone. Conversely, pretreatment with 0.5 mg/mL cherry for 1 h significantly increased COX-2 expression.

## 4. Discussion

The results of this study show that pretreating HAPI microglia cells with Montmorency tart cherry powder enhanced protection against oxidative and inflammatory stress by reducing stress-mediated signaling in a dose- and time-dependent manner. Because higher doses and longer treatment durations negatively affected cell viability and some stress-mediated signals, a concentration of between 0.125 and 0.25 mg/mL tart cherry, at a treatment time of 2 h, appeared to be the optimal combination.

Microglia mediate inflammation as a response to stress or injury in the central nervous system. This response can be beneficial, such as by recruiting bone marrow-derived cells to an injured brain region to aid in the healing process [30]. Alternatively, microglial activation can be problematic, due to the release of potentially neurotoxic substances [31]. Furthermore, inflammation in the central nervous system has been linked to autoimmune diseases such as multiple sclerosis [32] and central nervous system degenerative diseases [33], as well as accelerating disease progression and worsening symptoms [3]. Microglia, when in a highly activated state—such as that caused by stressors like LPS—produce inflammatory molecules such as cytokines, superoxide, and nitric oxide, ultimately leading to a cascade of pro-inflammatory proteins and cell death [34].

LPS application activates microglial cells, leading to the release of NO and TNF-α, which together can be neurotoxic [31]. NO is an inflammatory mediator released by iNOS. TNF-α is a cytokine which also mediates inflammation. Similarly, COX-2 is an enzyme that catalyzes the reaction that creates prostanoids, which in turn increase cellular inflammation [35]. LPS application to microglial cells increases the release of all four of these inflammatory mediators compared to cells not treated with LPS. However, significantly less NO was released from cells pretreated with doses of tart cherry powder ranging from 0.125 to 0.500 mg/mL for 1–4 h. The lower NO release at 0.500 mg/mL for 2 and 4 h, however, is likely due to decreased cell viability at these times and this concentration, because there are fewer cells that are present and therefore able to release NO. The shorter durations and lower concentrations of tart cherry did not cause cell death, but still lowered extracellular NO levels compared to controls not pretreated with cherry. Similarly, COX-2 expression was significantly reduced when pretreated with the lowest tart cherry doses for 2 h compared to cells treated with high doses of tart cherry powder or not treated at all. Extracellular TNF-α levels were also reduced for 0.062 and 0.125 mg/mL cherry at 1 h and at all concentrations at 2 h and 4 h. The 4-h pretreatment, however, was again too long a duration to pretreat the cells, and the lower TNF-α levels are most likely due to the decreased number of viable cells. The application of cherry powder did not significantly affect iNOS production in LPS-exposed cells. This result was unexpected, because iNOS produces NO, and NO levels were lowered by tart cherry application. The reason behind the reduction in NO production without concomitant reduction in iNOS is not clear, but one possibility is that tart cherry polyphenols could be affecting the activity of iNOS or the levels of its cofactors, rather than iNOS expression. Taken together, these results suggest that pretreatment with Montmorency tart cherry powder helps to reduce inflammation and OS in HAPI rat microglial cells after exposure to a known inflammatory substance in a dose- and time-dependent manner. Similar neuroprotective results have been seen with other food extracts, such as walnuts [36,37], blueberries [38], and açai [25].

Exposure to conditions that produce oxidative and inflammatory stress causes symptoms that mimic those traditionally seen in aging populations [39,40]. As humans age, our ability to defend against these substances and their effects weakens, putting elderly people at increased risk for neuronal disease and degradation [3]. Indeed, substantial deficits in cognitive and motor performance have been shown in older populations [41]. Therefore, oxidative stress and inflammation should be minimized in the brain to avoid these possible negative outcomes. One way to protect an aging brain against this damage is to curtail microglial activation with neuroprotective foods, such as tart cherries. Polyphenols are one class of food-derived chemicals that may protect microglia against detrimental activation [5,25]. In our model, the application of tart cherry powder to HAPI rat microglial cells provided access to the polyphenols from the cherries, which protected the cells against the deleterious effects of LPS, a known inducer of inflammation in microglia. Therefore, tart cherries (like other dark-colored fruits) may be effective in reducing inflammation and oxidative stress in the brain, thereby protecting against cognitive declines in aged populations. This protection might be one mechanism by which dietary supplementation of tart cherries to rats aged 19–21 months improved age-related deficits in behavioral and neuronal functioning [19]. Because of the known age-related consequences of inflammation and oxidative stress, it may be important to consume neuroprotective foods—such as those rich in polyphenols—to deter cognitive decline. These results, coupled with our previous studies, show that the addition of cherries to the diet may increase “health span” in aging, and may slow the aging process by reducing the incidence and/or delaying the onset of debilitating neurodegenerative disease.

## 5. Conclusions

In conclusion, Montmorency tart cherry powder, which is high in polyphenols, was effective in reducing inflammatory and oxidative stress signaling in HAPI rat microglial cells. Pretreatment with tart cherry decreased levels of LPS-induced NO, TNF-α, and COX-2 in a dose- and time-dependent manner versus those without pretreatment; the optimal combination was between 0.125 and 0.25 mg/mL tart cherry for 2 h. Therefore, tart cherries, like other dark-colored fruits, may be effective in reducing inflammatory and OS-mediated signals.
